# Association between baseline glycated hemoglobin level and atrial fibrillation recurrence following cryoballoon ablation among patients with and without diabetes

**DOI:** 10.1186/s12872-024-03784-4

**Published:** 2024-02-16

**Authors:** Zheng Chen, Ruixin Zhang, Xinlin Zhang, Wei Xu

**Affiliations:** 1 Department of Cardiology, Drum Tower Clinical College of Nanjing Medical University, No. 321, Zhongshan Road, Nanjing, 210008 China; 2 Department of Cardiology, Affiliated Drum Tower Hospital, Medical School of Nanjing University, No. 321, Zhongshan Road, Nanjing, 210008 China; 3 Cardiovascular Medicine, Baoshan Branch of Renji Hospital, Shanghai Jiao Tong University School of Medicine, Shanghai, China

**Keywords:** Atrial fibrillation, Cryoablation, Glucose, Glycated hemoglobin, Predictors

## Abstract

**Objectives:**

The study aims to assess the effect of baseline glycated hemoglobin (HbA1c) levels on atrial fibrillation (AF) recurrence following cryoballoon ablation in patients with and without diabetes.

**Methods:**

Consecutive AF patients receiving first cryoballoon ablation between April 2018 and April 2021 were included. AF recurrence and other clinical outcomes were recorded for a minimum of 12 months post-ablation, with regular assessments at 3, 6, and 12 months, followed by annual check-ups. The primary outcome was AF recurrence after ablation at longest follow-up. Multivariate Cox proportional hazards regression models were utilized to calculate the hazard ratio (HR) and 95% CI per standard deviation (SD) increase of baseline HbA1c level.

**Results:**

335 patients were included in the analysis. The mean age was 61.7 years, 61.8% were male. 12.8% had type 2 diabetes, and 81.7% of patients had paroxysmal AF. The median level of HbA1c was 5.3%, and the mean CHA_2_DS_2_-VAS_c_ score was 1.8. All cryoballoon ablation procedures, utilizing a 28-mm balloon, achieved successful pulmonary vein isolation. Over a median follow-up of 18 months, 105 patients (31.3%) experienced AF recurrence. In multivariate Cox proportional hazards analysis, a higher HbA1c level, persistent AF (HR 1.91, 95% CI 1.08 to 3.39, *P* = 0.026), alcohol consumption (HR 2.67, 95% CI 1.33 to 5.37, *P* = 0.006), and Nadir RSPV (HR 1.04, 95% CI 1.00 to 1.08, *P* = 0.005) were significant predictors of AF recurrence. Per-SD increase of HbA1c was associated with a 1.75-fold increase risk of AF recurrence (HR 1.75, 95% CI 1.39 to 2.21, *P* < 0.001). Subgroup analysis revealed that a higher HbA1c level was associated with a higher risk of AF recurrence in patients with and without diabetes, and in patients with paroxysmal and persistent AF.

**Conclusion:**

Baseline HbA1c level was an independent predictor of AF recurrence following cryoablation, both in patients with and without diabetes.

## Introduction

Atrial fibrillation (AF) is the most common arrhythmia in adults and is associated with complications such as ischemic stroke and heart failure. The global prevalence of AF has been increasing, and in China, it is expected to range from 5 to 6% in people over 80 years old [1]. In recent years, there has been growing recognition of the importance of lifestyle modifications in reducing the incidence and progression of AF. These modifications include weight loss, increased physical activity, limited alcohol intake, and control of glycemic levels and blood pressure [2–4].

The Framingham Study demonstrated a significant association between AF and diabetes mellitus (DM). DM increases the risk of AF development and is linked to factors such as left atrial enlargement, hyperinsulinemia, and chronic inflammation [5]. Emerging evidence suggest that sodium glucose cotransporter 2 inhibitors (SGLT2i), a type of glucose-lowering medications, may have a beneficial effect in reducing the risk of AF. Several studies have demonstrated an association between SGLT2i use and a lower risk of AF, indicating a potential primary preventive effect [6, 7].

Cryoballoon ablation, a procedure used to treat AF, has shown comparable success and safety to radiofrequency ablation. The second-generation cryoballoon has been found to be more effective in achieving pulmonary vein isolation (PVI) and is associated with equivalent or better clinical outcomes than the first-generation cryoballoon [8]. However, the effect of baseline glycemic level, optimally reflected by glycated hemoglobin (HbA1c), on AF recurrence after cryoballoon ablation is unknown. This study aims to explore the impact of baseline HbA1c levels on AF recurrence in patients undergoing cryoballoon ablation.

## Methods

### Study population

This study was a retrospective analysis of a prospective observational cohort study that included 335 consecutive patients with symptomatic paroxysmal or persistent AF. The study was conducted at Nanjing Drum Tower Hospital between April 2018 and April 2021. The main intervention in this study was PVI using the second-generation cryoballoon ablation technique. Patients aged ≤ 18 years, or with moderate-to-severe valvular stenosis or insufficiency were excluded, as were patients with any contraindication for PVI, such as the presence of acute thrombosis or a bleeding diathesis. Patients with a left atrium diameter (LAD) of ≥ 55 mm, those who had undergone previous AF ablation, and those with acute reversible causes of AF, including hyperthyroidism, were also excluded. The study was approved from the institutional ethics committee and was conducted in accordance with the principles outlined in the Declaration of Helsinki.

### Preprocedural management

In this study, all patients received uninterrupted anticoagulation treatment for at least four weeks before cryoballoon ablation procedure. For patients on warfarin, the procedure was performed if their international normalized ratio (INR) was maintained within the range of 2.0–3.0, indicating adequate anticoagulation. Antiarrhythmic therapy, except for β-blockers, was discontinued 4 to 5 half-lives prior to the PVI procedure. This was done to minimize the potential impact of antiarrhythmic medications on the outcomes of the ablation procedure.

Cardiac structure and function assessments were conducted using routine transthoracic echocardiography. This included evaluating parameters such as LAD and left ventricular ejection fraction (LVEF). Transesophageal echocardiography was performed before PVI to exclude the presence of intracavitary thrombi. Baseline fasting blood samples were collected from all patients on the first morning of their hospitalization.

### Cryoballoon ablation

The procedure was performed by 1 of 3 electrophysiologists (Zheng Chen, Xinlin Zhang and Wei Xu), all of whom have performed > 150 cryoballoon ablation procedures. The procedure was carried out using local anesthesia and deep sedation with fentanyl. A single transseptal puncture was performed using the BRK-1 needle and the standard 8.5-Fr SL1 long sheath from St. Jude Medical Inc. (MN, USA). Heparin (100 IU/Kg) was injected through a peripheral vein, and the activated clotting time (ACT) was monitored every 30 min to maintain it between 300 and 350s by administering additional heparin as needed. Pulmonary vein (PV) anatomy and left atrium were visualized using PV angiography.

Throughout the procedure, a cryoballoon with a size of 28 mm (Arctic Front, Medtronic Inc, MN, USA) and a circumferential mapping catheter with a size of 20 mm (Achieve, Medtronic, CA, USA) were consistently used. Following the PV angiography, the transseptal sheath was exchanged for a 15-Fr steerable sheath (FlexCath, Medtronic Inc, MN, USA) using a sniff guidewire. Through the steerable sheath, the 20-mm circumferential mapping catheter was placed proximal to each PV ostium to record PV signals before ablation.

The quality of PV occlusion was evaluated by the operator on a scale of 1–4, where 4 indicates a complete occlusion with no visible contrast leak into the LA [9]. Once acceptable vessel occlusion was achieved, a freeze-thaw cycle was initiated. The number and duration of cryoapplications for each PV ablation were at the physician’s discretion, typically targeting a total of 4 to 5 min based on the time to isolation (TTI). TTI, also known as time-to-effect, was recorded when the PV potentials disappeared or became dissociated from left atrium activity. To prevent phrenic nerve injury (PNI), the dacapolar catheter was positioned in the superior vena cava to stimulate the phrenic nerve through electrical pacing. Energy delivery was interrupted immediately if weakening or loss of diaphragmatic contraction was noted. If the AF rhythm persisted after the isolation of all PVs, sinus rhythm was restored through electrical cardioversion. The endpoint of cryoablation was defined as the bilateral electrical isolation of all PVs.

### Postprocedural management and follow-up

Post-procedure, patients were recommended to continue oral anticoagulation for a minimum of 3 months based on their CHAD_2_S_2_-VAS_c_ score. Additionally, antiarrhythmic drugs (AADs) were prescribed for all patients during the 3-month blanking period following the ablation, after which its discontinuation was strongly advised. All patients were followed up for a minimum of 12 months, with regular clinical assessments at 3-, 6-, and 12-month post-ablation, followed by annual check-ups. Monitoring of patients included electrocardiogram (ECG), Holter-ECG, and echocardiography to evaluate their cardiac status. Patients who experienced palpitations were advised to seek immediate medical attention at a nearby hospital. They were recommended to undergo an ECG test to detect any potential atrial arrhythmia. AF recurrence was defined as the presence of AF, atrial flutter, or atrial tachycardia lasting at least 30 s after the 3-month blanking period.

### Statistical analysis

Categorical variables were presented as numbers and percentages, while continuous variables were expressed as mean ± standard deviation (SD). The chi-square test was used to compare categorical variables, while the t-student test was performed to compare means for continuous variables. Cox proportional hazards regression models were utilized to calculate the HR and 95% CI per SD increase in the baseline HbA1c level, accounting for variables that exhibited a significant association with a *P* value < 0.1 in univariate analysis, as well as previously identified predictors from the literature. A sensitivity analysis was performed comparing baseline HbA1c levels ≥ the median (5.3%) to those < the median. Stepwise regression based on likelihood ratios was employed to construct multivariate prediction models for the time to recurrence after the ablation procedure. Subgroup analyses were performed in participants with and without diabetes at baseline, and in patients with paroxysmal or persistent AF. Statistical significance was defined as a *P* value < 0.05. All statistical analyses were conducted using STATA software version 10.0 (StataCorp) and SPSS version 25.0.

## Results

### Baseline characteristics

A total of 335 patients who underwent cryoballoon ablation were included in the analysis. 61.8% of patients were male, and the mean age was 61.7 ± 9.9 years. The mean CHA_2_DS_2_-VAS_c_ score was 1.8 ± 1.5, and 81.65% of patients had paroxysmal AF. The mean body mass index (BMI) was 25.3 ± 3.2, and the AF duration was 26.1 ± 33 months. The mean fasting blood glucose (FBG) level was 4.6 ± 0.66 mmol/L, and the median level of HbA1c was 5.3% (mean, 5.4%). The LAD was 40.9 ± 4.4 mm, and LVEF was 58.4 ± 4.1%. 12.8% of patients had type 2 diabetes at baseline, among which 14% were taking an SGLT2i (Table 1).

**Table 1:** Baseline characteristics of the study population

	Total	HbA1c ≥ median	HbA1c < median	*P* value
No.	335	183	152	
Age, years	61.7 ± 9.87	62.84 ± 8.77	60.33 ± 10.93	0.023
Sex				0.808
Male	207 (61.8%)	112 (61.2%)	95 (62.5%)	
Female	128 (38.2%)	71 (38.8%)	57 (37.5%)	
BMI	25.33 ± 3.18	25.37 ± 3.03	25.29 ± 3.37	0.818
AF type				0.097
Paroxysmal	273 (81.5%)	155 (84.7%)	118 (77.6%)	
Persistent	62 (18.5%)	28 (15.3%)	34 (22.4%)	
AF duration, months	26.1 ± 33	27.72 ± 32.95	24.15 ± 33.07	0.325
Smoking	35 (10.4%)	22 (12.0%)	13 (8.6%)	0.301
Alcohol habitus	31 (9.3%)	23 (12.6%)	8 (5.3%)	0.022
HTN	99 (29.6%)	42 (23.0%)	57 (37.5%)	0.004
T2DM	43 (12.8%)	27 (14.8%)	16 (10.5%)	0.249
Insulin	14 (4.2%)	9 (4.9%)	5 (3.3%)	0.587
SGLT2i	6 (1.8%)	3 (1.6%)	3 (2.0%)	0.999
CHF	12 (3.6%)	8 (4.4%)	4 (2.6%)	0.394
Stroke/TIA	33 (9.9%)	26 (14.2%)	7 (4.6%)	0.003
CHA_2_DS_2_-VAS_C_	1.78 ± 1.49	1.99 ± 1.56	1.52 ± 1.36	0.004
Dyslipidemia	63 (18.8%)	31 (16.9%)	32 (21.1%)	0.338
BNP, pg/mL	92.98 ± 94.6	93.86 ± 102	91.71 ± 83.22	0.858
FBG, mmol/L	4.61 ± 0.66	4.73 ± 0.82	4.46 ± 0.34	< 0.0001
HbA1c, %	5.41 ± 0.5	5.7 ± 0.52	5.06 ± 0.12	< 0.0001
LDL-C, mmol/L	2.45 ± 0.79	2.43 ± 0.79	2.48 ± 0.79	0.6
HDL-C, mmol/L	1.11 ± 0.23	1.14 ± 0.25	1.08 ± 0.18	0.015
TG, mmol/L	2.26 ± 15.02	2.96 ± 20.31	1.42 ± 0.55	0.352
UA, umol/L	338.79 ± 66.08	341.21 ± 76.79	335.87 ± 50.37	0.446
CREA, umolL	65.16 ± 14.82	66.55 ± 14.38	63.48 ± 15.21	0.059
eGFR, ml/min/1.73m^2^	105.17 ± 19.86	103 ± 18.63	108.47 ± 21.28	0.039
TSH, mIU/L	2.65 ± 1.62	2.71 ± 1.73	2.58 ± 1.48	0.483
FT3, pmol/L	4.75 ± 0.6	4.75 ± 0.58	4.75 ± 0.63	0.994
FT4, pmol/L	16.93 ± 2.2	16.97 ± 2.21	16.88 ± 2.19	0.707
CRP, mg/L	4.74 ± 8.79	4.96 ± 10.81	4.39 ± 4.15	0.628
Hb, g/L	139.84 ± 15.94	140.09 ± 16.05	139.43 ± 15.85	0.761
LAD, mm	40.92 ± 4.38	41.36 ± 3.77	40.37 ± 4.99	0.042
LVEF, %	58.35 ± 4.11	58.45 ± 3.91	58.18 ± 4.43	0.635
IVSTD, mm	9.24 ± 1.99	9.09 ± 1.19	9.41 ± 2.65	0.142
LVPWTD, mm	8.91 ± 1.03	8.89 ± 0.95	8.93 ± 1.11	0.739
LVDD, mm	49.51 ± 5.46	49.46 ± 6.74	49.57 ± 3.29	0.857
PAP, mmHg	26.32 ± 9.36	25.58 ± 10.13	27.48 ± 7.92	0.138

Data expressed as number, mean standard deviation or median (interquartile range)

* *p* < 0.05 (significant difference between groups)

BMI, body mass index; AF, atrial fibrillation; HTN, hypertension; T2DM, type 2 diabetes mellitus; CHF, chronic congestive heart failure; TIA, transient ischemic attack; FBG, fasting blood glucose; HbA1c, Glycosylated hemoglobin; LDL-C, low-density lipoprotein cholesterol; HDL-C, high-density lipoprotein cholesterol; TG, triglyceride; UA, uric acid; CREA, creatinine; eGFR, estimated glomerular filtration rate; TSH, thyroid stimulating hormone; FT3, free triiodothyronine; FT4, free thyroxine; CRP, C-­reactive protein; Hb, hemoglobin; LAD, left atrial diameter; LVEF, left ventricular ejection fraction; IVSTD, interventricular septal thickness dimensions; LVPWTD, left ventricular posterior wall dimensions; LVDD, left ventricular diastolic diameter; PAP, pulmonary artery pressure; SGLT2i, sodium glucose cotransporter 2 inhibitor; TIA, transient ischemic attack

All cryoballoon ablation procedures were performed with a large 28-mm balloon and PVI was successfully achieved in all veins. A Grade 4 occlusion score was achieved in 95.1% of veins (1314 of 1382 veins) and a Grade 3 or above was achieved in 100% of veins (1382 of 1382). Mean procedural time and mean fluoroscopy time were 107.8 ± 35.8 and 25.6 ± 16.8 min, respectively. The TTI was 41.3 ± 16.6 s for the left superior pulmonary vein (LSPV), 31.2 ± 15.9 s for the left inferior pulmonary vein (LIPV), 36.0 ± 23.0 s for the right inferior pulmonary vein (RIPV), and 33.1 ± 20.2 s for the right superior pulmonary vein (RSPV). The minimal temperature achieved was − 49.7 ± 5.2 °C during LSPV ablation, − 44.6 ± 4.9 °C during LIPV ablation, − 46.6 ± 8.7 °C during RIPV ablation, and − 52.2 ± 4.9 °C during RSPV ablation. Right median pulmonary vein (RMPV) ablation was performed in 42 (12.5%) patients (Table 2). 114 premature abortions of the freeze cycle were documented in all patients, due to suboptimal occlusion or TTI, or PNI.

**Table 2:** Procedure-related characteristics

	Total	HbA1c ≥ median	HbA1c < median	*P* value
Time_LSPV, s	302.77 ± 67.2	308.84 ± 60.37	295.57 ± 74.05	0.078
ThawTime_LSPV, s	41.54 ± 9.97	41.04 ± 8.94	42.11 ± 11.09	0.525
Nadir_LSPV, ℃	-49.72 ± 5.15	-49.46 ± 4.99	-50.02 ± 5.35	0.326
TTI_LSPV, s	41.27 ± 16.59	42.19 ± 16.74	40.23 ± 16.49	0.477
Time_LIPV, s	289.91 ± 51.68	296.69 ± 51.97	281.78 ± 50.32	0.009
ThawTime_LIPV, s	34.7 ± 8.66	34.45 ± 7.47	34.98 ± 9.92	0.727
Nadir_LIPV, ℃	-44.56 ± 4.99	-44.48 ± 5.24	-44.66 ± 4.68	0.74
TTI_LIPV, s	31.18 ± 15.87	33.07 ± 19.92	29.04 ± 9.12	0.166
Time_RSPV, s	258.97 ± 68.79	259.47 ± 62.38	258.37 ± 75.9	0.885
ThawTime_RSPV, s	47.46 ± 11.96	48.23 ± 11.49	46.59 ± 12.51	0.417
Nadir_RSPV, ℃	-52.22 ± 4.97	-52.05 ± 5.48	-52.42 ± 4.29	0.496
TTI_RSPV, s	33.09 ± 20.21	34.46 ± 24.18	31.33 ± 13.64	0.41
Time_RIPV, s	269 ± 65.52	273.76 ± 61.95	263.46 ± 69.24	0.156
ThawTime_RIPV, s	35.35 ± 11.74	34.34 ± 9.33	36.48 ± 13.95	0.293
Nadir_RIPV, ℃	-46.6 ± 8.69	-46.92 ± 7.09	-46.24 ± 10.26	0.478
TTI_RIPV, s	35.98 ± 23	41.3 ± 21.21	30.24 ± 23.88	0.083
Time_RMPV, s	188 ± 64.97	191.09 ± 70.45	184.26 ± 59.33	0.739
ThawTime_RMPV, s	36.56 ± 11.27	33.15 ± 11.17	40.25 ± 10.59	0.117
Nadir_RMPV, ℃	-45.55 ± 6.41	-45.78 ± 5.66	-45.26 ± 7.36	0.797
TTI_RMPV, s	27.5 ± 17.59	26 ± 19.81	30 ± 16.82	0.781
RMPV ablation	42 (12.5%)	23 (54.8%)	19 (45.2%)	0.985
Procedural time, min	107.8 ± 35.8	108.2 ± 34.6	106.9 ± 35.2	0.871
Fluoroscopy time, min	25.6 ± 16.8	25.4 ± 15.9	25.1 ± 16.2	0.798

Data are expressed as mean ± SD or n (%)

LSPV, left superior pulmonary vein; LIPV, left inferior pulmonary vein; RSPV, right superior pulmonary vein; RIPV, right inferior pulmonary vein; RMPV, right middle pulmonary vein; TTI, time to isolation

### Baseline differences across HbA1c classes

Among the patients, 183 had an HbA1c level ≥ the median (5.3%). Patients with a higher HbA1c tended to be older (62.8 vs. 60.3 years), have a lower percentage of hypertension (23.0% vs. 37.5%), a higher percentage of stroke/transient ischemic attack (14.2% vs. 4.6%), had a higher CHA_2_DS_2_-VAS_c_ score (1.99 vs. 1.52), HDL-C levels (1.14 vs. 1.08 mmol/L), LAD (41.4 vs. 40.4 mm), and a lower estimated glomerular filtration rate (103.0 vs. 108.5 ml/min/1.73m^2^). The percentage of patients with diabetes, persistent atrial fibrillation, and the mean duration of AF were not statistically different between groups (Table 1). Patients with a higher HbA1c had a higher Time LIPV compared to the other group, while other procedural characteristics were similar (Table 2).

### Complications

There were no deaths, atrioesophageal fistulas, or pericardial effusions/tamponades during the periprocedural period. A single case of stroke was diagnosed four hours after an ablation procedure. Three significant complications at the groin site were identified, comprising two femoral pseudoaneurysms and one arteriovenous fistula. Surgical closure was required for one pseudoaneurysm and the arteriovenous fistula. Hematemesis occurred in three patients, all of which were mild and resolved within two days. A total of 11 PNIs were observed, all occurring during RSPV cryoablation in these 11 patients. With the exception of one asymptomatic event, all PNIs resolved before 3 months (Table 3).

**Table 3:** Serious procedure-related complications

Serious procedure-related complications	Total	HbA1c ≥ median	HbA1c < median
Groin-site complication†	3 (0.9%)	1 (0.55%)	2 (1.3%)
Phrenic nerve injury	11 (3.3%)	6 (3.3%)	5 (3.3%)
Cardiac tamponade or pericardial effusion	0 (0)	0 (0)	0 (0)
Hematemesis	3 (0.9%)	2 (1.1%)	1 (0.66%)
Stroke or TIA	1 (0.3%)	0 (0)	1 (0.66%)

Data expressed as number (percentage)

TIA, transient ischemic attack

†Vascular pseudoaneurysm or arteriovenous fistula

### Efficacy outcomes

After the blanking period, 19 patients received AAD therapy as follows: amiodarone (*n* = 5), dronedarone (*n* = 2) and propafenone (*n* = 12). During a median follow-up of 18 months, 105 patients (31.3% of the total) experienced AF recurrence. In univariate analysis, a higher recurrence rate was observed in patients with a higher HbA1c and FBG, persistent AF, a larger LAD, a longer AF duration, a higher Nadir_RSPV, and in patients who smoked or with an alcohol habit. In the multivariate Cox proportional hazards analysis, a higher HbA1c level, persistent AF (HR 1.91, 95% CI 1.08 to 3.39, *P* = 0.026), alcohol consumption (HR 2.67, 95% CI 1.33 to 5.37, *P* = 0.006) and Nadir RSPV (HR 1.04, 95% CI 1.00 to 1.08, *P* = 0.05) remained significant predictors of AF recurrence (Table 4). Per-SD increase of HbA1c was associated with a 1.75-fold increase risk of AF recurrence (HR 1.75, 95% CI 1.39 to 2.21, *P* < 0.001). Furthermore, when categorizing patients based on the median level of HbA1c, an increased AF recurrence rate was observed in patients with HbA1c levels ≥ the median (HR 2.99, 95% CI 1.44 to 3.7, *P* = 0.001) (Table 5).

**Table 4:** Cox regression analysis for predictors of atrial fibrillation recurrence

	Univariate analysis		Multivariate analysis	
	HR (95% CI)	*P* value	HR (95% CI)	*P* value
Age	1.01 (0.98, 1.03)	0.673	1.00 (0.98, 1.03)	0.835
Sex	1.18 (0.74, 1.90)	0.485	1.18 (0.76, 1.85)	0.443
BMI	1.04 (0.96, 1.12)	0.333		
AF duration	1.01 (1.00, 1.01)	0.074	1.005 (1.00, 1.01)	0.108
Persistent AF	1.82 (1.07, 3.11)	0.028	1.91 (1.08, 3.39)	0.026
HTN	1.11 (0.70, 1.77)	0.667		
CHF	0.72 (0.20, 2.72)	0.631		
Stroke/TIA	0.68 (0.29, 1.55)	0.357		
CHA_2_DS_2_-VAS_C_	1.09 (0.94, 1.27)	0.262		
eGFR	1.00 (0.98, 1.01)	0.643		
Hb	1.01 (0.99, 1.02)	0.562		
LA diameter	1.08 (1.02, 1.15)	0.009	1.03 (0.97, 1.11)	0.309
LVEF	0.98 (0.92, 1.05)	0.577		
Smoking	2.96 (1.45, 6.01)	0.003	1.25 (0.61, 2.57)	0.541
Alcohol habitus	5.5 (2.49, 12.17)	< 0.001	2.67 (1.33, 5.37)	0.006
Dyslipidemia	1.58 (0.90, 2.79)	0.115		
BNP	1.00 (1.00, 1.01)	0.128		
TSH	1.03 (0.89, 1.18)	0.72		
FBG	2.22 (1.46, 3.38)	< 0.0001	1.00 (0.72, 1.39)	0.999
HbA1c per SD increase	2.67 (1.92, 3.71)	< 0.0001	1.75 (1.39, 2.21)	< 0.001
hsCRP	1.01 (0.98, 1.04)	0.581		
PAP	1.02 (0.99, 1.05)	0.291		
Time_RSPV	1.00 (0.99, 1.00)	0.152		
ThawTime_LSPV	1.02 (0.98, 1.06)	0.402		
Nadir_LSPV	1.01 (0.97, 1.06)	0.598		
TTI_LSPV	1.02 (1.00, 1.04)	0.124		
Time_LIPV	1.00 (1.00, 1.01)	0.8		
ThawTime_LIPV	1.03 (0.99, 1.08)	0.161		
Nadir_LIPV	1.01 (0.97, 1.06)	0.598		
TTI_LIPV	1.01 (0.99, 1.04)	0.395		
Time_RSPV	1.00 (0.99, 1.00)	0.762		
ThawTime_RSPV	1.00 (0.97, 1.04)	0.849		
Nadir_RSPV	1.06 (1.02, 1.11)	0.008	1.04 (1.00, 1.08)	0.05
TTI_RSPV	0.995 (0.97, 1.02)	0.632		
Time_RIPV	1.00 (1.00, 1.01)	0.3		
ThawTime_RIPV	0.99 (0.96, 1.03)	0.739		
Nadir_RIPV	1.01 (0.98, 1.03)	0.661		
TTI_RIPV	1.01 (0.98, 1.03)	0.685		
Time_RMPV	1.00 (0.99, 1.01)	0.597		
ThawTime_RMPV	1.03 (0.94, 1.12)	0.583		
Nadir_RMPV	1.03 (0.92, 1.14)	0.657		
TTI_RMPV	0.67 (0.37, 1.22)	0.19		
RMPV ablation	0.75 (0.36, 1.56)	0.443		

Abbreviations as in Tables 1 and 2

**Table 5:** HR estimates and 95% CI for the association between HbA1c and the risk of AF recurrence, by diabetes status and AF types

	No	HR (95% CI) per-SD increase of HbA1c	*P* value	HR (95% CI) HbA1c ≥ median vs. <median	*P* value
Overall	335	1.75 (1.39, 2.21)	< 0.001	2.29 (1.44, 3.7)	0.001
T2DM	43	1.89 (1.26, 2.83)	0.002	3.45 (1.21, 9.84)	0.02
Non T2DM	292	2.18 (1.56, 3.03)	< 0.001	2.5 (1.43, 4.35)	0.001
Paroxysmal AF	273	1.8 (1.27, 2.56)	0.001	1.91 (1.13, 3.24)	0.016
Persistent AF	62	2.2 (1.46, 3.3)	< 0.001	6.27 (1.94, 20.2)	0.002

AF, atrial fibrillation; HR, hazard ratio; SD, standard deviation; T2DM, type 2 diabetes mellitus;

We also performed multivariate Cox proportional hazards analysis to identify independent variables associated with AF recurrence in patients both with and without diabetes, as well as in patients with paroxysmal and persistent AF. Across all subgroups, an elevated HbA1c level was consistently linked to a heightened risk of AF recurrence, whether analyzed per SD increase or categorized based on the median level (Table 5).

## Discussions

To the best of our knowledge, this study represents the first attempt to assess the predictive impact of glucose levels, as indicated by HbA1c levels, on the risk of AF recurrence following cryoballoon ablation. Our investigation, encompassing 335 patients who underwent initial ablation procedures with close follow-up, unveiled a noteworthy predictive influence of baseline HbA1c levels. This effect was evident whether analyzed per standard deviation increase or categorized based on the median level. Of the participants, 13% had diabetes at baseline, and our analysis confirmed a consistent prognostic effect of HbA1c in both diabetic and non-diabetic patients. Similar outcomes were also observed in patients with paroxysmal or persistent AF. Our data emphasize the significance of monitoring or controlling baseline glucose levels to mitigate AF recurrence risk in both diabetic and non-diabetic individuals. Furthermore, our findings suggest that integrating glycemic monitoring and management into post-cryoablation AF treatment might enhance long-term outcomes.

Diabetes is a prevalent chronic condition that significantly contributes to cardiovascular disease, increasing the risk of cardiovascular incidents and mortality [10]. It is well-established that diabetes elevates the risk of AF, which can exacerbate clinical symptoms, diminish quality of life, and lead to more frequent hospitalizations and higher mortality rates, often due to embolic complications associated with AF [11]. The landmark Framingham Heart Study was among the first to identify a heightened risk of AF in individuals with diabetes [5]. Subsequent research by Dubin and colleagues revealed that poor glycemic control and a longer duration of diabetes are associated with an increased incidence of AF [12]. This association was corroborated by a meta-analysis that reported a 34% higher risk of AF in diabetic individuals [13], a finding further supported by another meta-analysis in 2017 which confirmed a significant link between elevated HbA1c levels and the occurrence of AF in prospective cohort studies [14].

Pre-diabetes, characterized by elevated blood glucose levels not yet in the diabetic range, can still lead to chronic inflammation, endothelial damage, and insulin resistance. Research has consistently shown that HbA1c levels correlate with the risk of AF. A 2020 meta-analysis highlighted that an HbA1c level above 6.3% significantly increases AF risk, regardless of diabetes diagnosis or patient awareness [15]. Furthermore, each 20 mg/dl rise in blood glucose corresponds to a 10% increase in AF incidence [16]. A large-scale study involving over two million subjects identified a marked increase in AF risk associated with higher HbA1c levels, in both diabetic and non-diabetic individuals [17]. Therefore, monitoring HbA1c levels may be a valuable tool for predicting AF, especially in those with pre-diabetes or diabetes.

Several mechanisms might explain the link between high HbA1c levels and AF. Persistent elevation of HbA1c can lead to the formation of advanced glycosylation end products (AGEs), contributing to left atrial fibrosis and strain, thus promoting AF development [18]. Additionally, in those with pre-diabetes, metabolic disturbances or hyperglycemia may increase atrial conduction time and P-wave dispersion, which are known to elevate AF risk in otherwise healthy individuals [19, 20]. These factors collectively may amplify the propensity for AF.

New-generation SGLT2i have shown efficacy beyond blood glucose control, including reductions in blood pressure, weight, and markers of oxidative stress and inflammation—all key factors in the pathogenesis of AF. In patients with type 2 diabetes, the DECLARE-TIMI 58 study revealed that SGLT2i significantly decreased the occurrence of atrial tachyarrhythmias [7]. This finding is echoed by recent research suggesting SGLT2i may reduce AF recurrence post-AF ablation in diabetic patients, with further support from independent studies [21–23]. Although the exact pathways remain unclear, SGLT2i likely intervenes in various stages of AF risk, potentially preventing changes in atrial electrical properties and enhancing cellular metabolism [24].

Catheter ablation has outperformed AADs in sustaining sinus rhythm for AF patients, as confirmed by numerous randomized controlled trials. However, post-procedure recurrence rates remain high, affecting 20–40% of patients [16]. Studies focusing on diabetic patients undergoing radiofrequency ablation for AF have found a direct correlation between baseline glucose levels and recurrence of atrial arrhythmias [25]. For example, a greater than 10% improvement in HbA1c levels within a year prior to ablation was associated with a notable reduction of 30% in AF recurrence [26]. Similarly, HbA1c levels ≥ 6.5% have been predictive of recurrent atrial arrhythmias and increased cardiovascular hospitalizations [27]. Moreover, Lu et al. reported that patients achieving HbA1c levels below 6.9% had a better success rate in AF ablation [28]. These findings underscore the importance of HbA1c as a marker in the management of AF treatments, including the efficacy of radiofrequency ablation in patients with concurrent AF and type 2 diabetes.

PVI remains the cornerstone treatment for AF [29], with both radiofrequency catheter ablation and cryoballoon ablation showing efficacy for paroxysmal AF [30]. Cryoballoon ablation, in particular, offers advantages like reduced procedure time and a steeper learning curve [31]. Until now, the influence of glucose levels on outcomes post-cryoballoon ablation, especially in non-diabetic patients, has not been examined. We found that in both diabetic and non-diabetic patients, higher HbA1c was associated with an increased risk of recurrence after cryoablation. The baseline HbA1c level may not only represent the average glucose level before ablation but could also be indicative of the post-ablation glucose levels, given the absence of specific interventions targeting glucose post-ablation. Therefore, it is plausible that a patient’s regular glucose levels could influence AF recurrence following ablation. Consequently, it is prudent to consider lowering HbA1c levels several months before ablation, especially in individuals scheduled for selective ablation procedures. Sustaining a lower HbA1c level is advisable to mitigate the risk of AF recurrence after cryoballoon ablation. Adopting a healthy diet, maintaining regular exercise, and managing body weight might be beneficial for all AF patients. In addition, a more stringent glucose-lowering target may be appropriate to minimize AF recurrence for those with diabetes. Nevertheless, further evidence from extensive prospective studies is essential to validate these findings and determine the optimal HbA1c target for diabetic patients. Future clinical trials are also essential to evaluate the potential of glucose-lowering agents, beyond SGLT2 inhibitors, to decrease the recurrence of AF post-ablation in diabetic patients. Our observations indicated a heightened recurrence rate in patients with a history of alcohol consumption, implying that discontinuation of alcohol intake could potentially benefit individuals with AF by reducing the risk of AF recurrence. A noteworthy discovery from our study was the association between a higher Nadir_RSPV and increased AF recurrence, the underlying mechanism of which remains unclear. It is conceivable that a lower Nadir_RSPV may lead to superior vena cava (SVC) isolation or potential conduction delay in the SVC during cryoballoon ablation of the RSPV [32, 33].

The study has certain limitations, including its relatively short-term follow-up duration, the possibility of unadjusted potential confounding factors, and the absence of longer-term or continuous AF recording using implantable cardiac monitors or a 1-week Holter-ECG.

## Conclusions

Baseline HbA1c level was independent predictor of AF recurrence following cryoablation, both in patients with and without diabetes.

## Data Availability

No datasets were generated or analysed during the current study.
